# A 4pL item response theory examination of perceived stigma in the screening of eating disorders with the SCOFF among college students

**DOI:** 10.1007/s40519-023-01604-w

**Published:** 2023-10-04

**Authors:** Lucy Barnard-Brak, Zhanxia Yang

**Affiliations:** 1 University of Alabama, P.O. Box 870232, Tuscaloosa, AL 35487 USA; 2 Boston College, 140 Commonwealth Ave, Chestnut Hill, MA 02467 USA

**Keywords:** Eating disorders, Perceived stigma, SCOFF, Item response theory, IRT

## Abstract

We examined the psychometric properties of the SCOFF, a screening instrument for eating disorders, with consideration of the perceived stigma of items that can produce socially desirable responding among a sample of college students. The results of the current study suggest evidence of the sufficient psychometric properties of the SCOFF in terms of confirmatory factor and item response theory analyses. However, two items of the SCOFF revealed that individuals who otherwise endorsed other items of the SCOFF were less likely to endorse the items of Fat and Food. It is hypothesized that this is the result of perceived stigma regarding those two items that prompts individuals to respond in a socially desirable way. A weighted scoring procedure was developed to counteract the performance of these two items, but the psychometric performance was only slightly better and there would be a clear tradeoff of specificity over sensitivity if utilized. Future research should consider other ways to counteract such perceived stigma.

*Level of evidence* Level III: Evidence obtained from cohort or case–control analytic studies.

## Introduction

The early detection and diagnosis of eating disorders is considered important given the long-term and lifelong consequences of these disorders [43]. Fukutomi et al. [20] found that the earlier interventions and treatments were associated with a higher rate of recovery from an eating disorder. Eating disorders such as Anorexia Nervosa, Bulimia Nervosa, Binge Eating Disorder, Pica, and Avoidant/Restrictive Food Intake Disorder per the DSM-5-TR [1] would all appear to potentially benefit from this early detection and diagnosis. College students as young adults may be considered as more at risk of eating disorders than the population at large [16, 24, 34, 47]. In addition, college students who may be at risk of eating disorders have been found to be more subsequently at risk for suicide [34], psychosis symptoms [23], and depression and anxiety [29]. Several factors have been associated with the development of eating disorders for the college student population in particular such as loneliness [22], food insecurity [7], as body dysmorphia and the use performance enhancing drugs [23]. The current study focuses on this young adult population in assessing risk of eating disorders.

To screen for the early detection of eating disorders, the SCOFF (Sick, Control, One Stone, Fat, and Food; [38] presents one of the most widely used early screening measures for eating disorders in community-based settings [30] and in epidemiological research [48]. The SCOFF screening instrument represents a quick and relatively accurate means of screening for eating disorders with five, dichotomous response items [11, 30, 46]. Each of the five items correspond to one of the letters of the SCOFF. The ‘S’ as the first item refers to sick with the item stating, “Do you make yourself **S**ick because you feel uncomfortably full?” [38], p. 1467. The ‘C’ in the second item refers to control with the item stating, “Do you worry you have lost **C**ontrol over how much you eat?” [38], p. 1467. The ‘O’ for the third item refers to one stone referencing its British origins with the item stating, “Have you recently lost more than **O**ne stone (6.35 kg or 14 pounds) in a three-month period?” [38], p. 1467. The first ‘F’ in the fourth item refers to fat with the item stating, “Do you believe yourself to be **F**at when others say you are too thin?” [38], p. 1467. The second ‘F’ in the fifth item refers to food with the item stating, “Would you say **F**ood dominates your life?” [38], p. 1467.

The SCOFF has been translated into several languages and has been examined internationally for its psychometric properties for over the past twenty years overall positively [3, 8, 32, 42] with some mixed evidence [48]. While many screening instruments for eating disorders exist, the overall body of literature provides evidence that supports the reliability and validity of the SCOFF given its status as a brief screener with ‘yes’ versus ‘no’ responses [12, 43]. In a meta-analysis of its diagnostic accuracy via Receiver Operating Curve (ROC) studies, Botella et al. [12] concluded that the SCOFF would be a highly recommended screening instrument for eating disorders. As a result, the U.S. Preventive Services Task Force indicated the SCOFF as having, “adequate adequacy for detecting eating disorders,” [18], p. 1068.

The purpose of the current study was to examine the psychometric properties of the SCOFF, a screening instrument for eating disorders, with consideration of the perceived stigma of items that can produce socially desirable responding. The psychometric properties examined in the current study included the construct validity via confirmatory factor and item response theory analyses as well as criterion validity via ROC analyses and reliability via Cronbach’s alpha values. Anonymous, self-reported diagnoses of eating disorders would appear to have more value as having less stigma or social desirability concerns by respondents [43]. To achieve this purpose, we utilized item response theory (IRT) techniques to estimate the degree of perceived stigma that can result in pseudo-social desirability via a four parameter logistic (pL) IRT model. After determining the influence of this parameter, we adjust SCOFF scoring accordingly and then examine the relative performance of the SCOFF as originally scored versus the adjusted SCOFF scores in screening for eating disorders as self-reported by respondents.

To examine these psychometric properties of the SCOFF, item response theory techniques were utilized to examine the construct validity. IRT techniques were especially utilized given the dichotomous response format (i.e., ‘yes’ versus ‘no’) of the items [9, 10]. These item response theory (IRT) examinations have been worthwhile but (1) have been limited to adolescents and (2) have not fully explored the issues of perceived stigma and thus social desirability in reporting symptoms of eating disorders. In particular, the fourth parameter of the upper asymptote can be utilized via IRT techniques to estimate the degree of pseudo-social desirability in item responding depending upon the item. We refer to this as pseudo-social desirability as these analyses cannot unequivocally determine that this is response pattern is the result of perceived stigma in much the way that the third parameter of the lower asymptote is referred to as pseudo-guessing [11, 49]. ROC curve analyses were used to establish evidence of criterion validity of the SCOFF by examining the ability of screening instrument scores to accurately identify individuals with self-reported eating disorders [51].

## Methods

### Sample

 The sample consisted of 89,181 individuals who participated in the 2019–2020 Healthy Minds Study (HMS) data collection [25]. The Healthy Minds Study surveyed college students at seventy-five institutions of higher education across the United States. The mean age of participants was 23.113 years (*SD* = 6.592). The mean body mass index (BMI) value for the sample was 25.868 (*SD* = 6.361). As the sample was somewhat skewed, we report the median BMI value of 24.392 as well. These BMI values were calculated based upon the self-reported values of height and weight. Table 1 provides the descriptive statistics in terms of gender as well as race/ethnicity for the sample.

**Table 1:** Descriptive statistics for sample

Gender	
Male	30.332% (*n* = 27,051)
Female	67.389% (*n* = 60,099)
Trans male/Trans man	0.292% (*n* = 260)
Trans female/Trans woman	0.137% (*n* = 122)
Gender queer or gender non-conforming	1.153% (*n* = 1028)
Other	0.574% (*n* = 512)
Race/Ethnicity	
African American	8.440% (*n* = 7527)
American Indian or Alaska Native	1.510% (*n* = 1347)
Asian American/Asian	13.166% (*n* = 11,742)
Hispanic/Latino/a	12.032% (*n* = 10,730)
Native Hawaiian or Pacific Islander	0.660% (*n* = 589)
Middle Eastern, Arab, or Arab American	2.256% (*n* = 2012)
White	70.544% (*n* = 62,912)
Other	1.538% (*n* = 1372)

### Measures

The SCOFF is a five-item scale with dichotomous response format of ‘yes’ (= 1) versus ‘no’ (= 0). The SCOFF is scored by summing up the ‘yes’ responses. A score of at least 2 is the recommended cutoff score for the SCOFF indicating the likelihood of an eating disorder being present [38]. For the sample, the mean score was 0.934 (*SD* = 1.150) with scores ranging from 0 to 5. Participants were also asked to self-report diagnoses of eating disorders via the following question, “Specifically, which of the following eating disorders were you diagnosed with by a professional?” [25], p. 60). This question implies that the diagnosis was current as potentially ongoing but participants could have been interpreted as a lifetime diagnosis only. The preceding question was, “Have you ever been diagnosed with any of the following conditions by a health professional (e.g., primary care doctor, psychiatrist, psychologist, etc.)? (Select all that apply),” which implies both lifetime and current status with the use of word, “ever.” Within the HMS, participants had the option to self-report the following eating disorders: Anorexia Nervosa, 1.643% (*n* = 1466); Bulimia Nervosa, 0.967% (*n* = 862); Binge-Eating Disorder, 0.719% (*n* = 641); Pica, less than 0.1% (*n* = 25); Avoidant/Restrictive Food Intake Disorder, 0.8% (*n* = 685); and Other, 0.028% (*n* = 208). We also aggregated these values, which indicated that approximately 3.271% (*n* = 2917) reported at least one eating disorder.

### Analyses

Analyses were conducted in M*plus* (*v*. 8.1; [40]) and MedCalc (*v*. 20.106; [37]). Approximately 8.402% of the data were missing on the SCOFF metric. Missing data were handled via full information maximum likelihood. First, we examined for the unidimensionality of the construct via confirmatory factor analyses. A statistically significant Chi-square (χ^2^) statistic may be indicative of unacceptable model yet other model fit statistics such as the Comparative Fit Index (CFI), Tucker Lewis Index (TLI), and the Root Mean Square Error of Approximation (RMSEA). CFI and TLI values of 0.950 and better indicate acceptable fit while RMSEA values at or less than 0.080 also indicates acceptable fit (Little 2013). We calculated the internal consistency of scores for the data obtained via Cronbach’s alpha, in which scores at or greater than 0.700 may be considered as acceptable (Little 2013). Second, we examined for local independence by comparing models via Akaike Information Criterion (AIC), Bayesian Information Criterion (BIC), and sample size adjusted Bayesian Information Criterion (BIC_SS_). Lower values of AIC, BIC, and BIC_SS_ indicate better model fit relative to each other. After establishing unidimensionality and local independence, we employed Item Response Theory (IRT) techniques. IRT provides for the estimation of up to four item parameters along a continuum of the latent construct as measured as theta (θ): item discrimination (*a*); item difficulty (*b*); item guessing (*c*); and item carelessness (*d*). To determine the number of parameters to be estimated, we will compare 2 parameter logistic (pL; *a* & *b* parameters estimated), 3pL (*a*, *b*, & *c* parameters estimated), and 4pL (*a*, *b*, *c*, & *d* parameters estimated) models via AIC, BIC, and BIC_SS_ values.

Item discrimination (*a*) parameter values represented the slope of the item characteristic curve, where the individual has a 50% probability of endorsing (i.e., the point of inflection) with values of at least 0.40 to indicate low but acceptable item discrimination with higher values being more desirable [5, 15]. For item difficulty (*b*) values, a range of values of is often desired to measure the continuum of the construct, typically values ranging from 3.000 to − 3.000 [4, 5]. Item pseudo-guessing (*c*) values at the lower asymptote (or *y*-intercept) should be low and not exceed 0.20 [49]. This guessing occurs when individuals who otherwise score low on a construct then correctly endorse an item, which would be unexpected. In the context of the SCOFF, there is no correct versus incorrect response, thus an individual endorsing an item when they do not endorse other items would indicate confusion about that item. Item response theory is often used in achievement testing where there are clear right/correct or wrong/incorrect answers. In mathematics, unless the item is a poorly worded word problem, there should be no confusion by what is meant by 12 + 3 =? for instance. In psychological constructs, there can be different interpretations of the same item revealing confusing among participants. For the third parameter of pseudo guessing, individuals with even a low degree of symptoms may endorse an item when interpreting it differently. Item carelessness or slip (*d*) values should be high at least 0.900 [50]. These slips occur when individuals who otherwise score high on a construct fail to endorse an item that they should. This may be due to a variety of reasons. Carelessness is one characterization for this occurrence, or the item may trigger individuals as experiencing perceived stigma, thus individuals may respond in a socially desirable way. Carelessness in the case of achievement items would be when a student who is getting 90% of items correct misses an item that they should answered correctly based upon the difficulty of the item. In this instance of achievement, the student was simply careless to miss the item. This carelesssness parameter, in the context of a psychological disorder would tend to manifest as social desirability. An individual who would have an otherwise high score but for one item that they did not endorse because they may not have wanted to admit to that item to themselves or others given societal expectations. Thus, in the current study, this fourth parameter of slip was referred to as pseudo-social desirability. Pseudo in the sense that this social desirability cannot be verified but is inferred in much the same way that guessing is inferred, thus termed pseudo-guessing [49]. We also examined for gender differences that may be statistically significant via differential item functioning analyses given past research indicating the presence of gender differences [19, 21, 26, 41].

Finally, Receiver Operator Characteristic (ROC) curve analyses were calculated to determine model fit in terms of diagnostic accuracy using the DeLong, DeLong, and Clarke-Pearson [14] method. In ROC curve analyses, the relationship of sensitivity over 1- specificity is analyzed, which produces an area under curve that is estimated. Area under the curve (AUC) values of 0.700 and greater indicate acceptable fit [17]. Sensitivity was calculated as the number of true positives (i.e., positive screener with diagnosis) divided by the sum of true positives and false negatives (i.e., negative screener without diagnosis) [51]. Specificity was calculated as the number of true negatives (i.e., negative screener with a diagnosis) divided by the sum of true negatives and false positives (i.e., positive screener without diagnosis) [51].

## Results

**Fig. 1:**
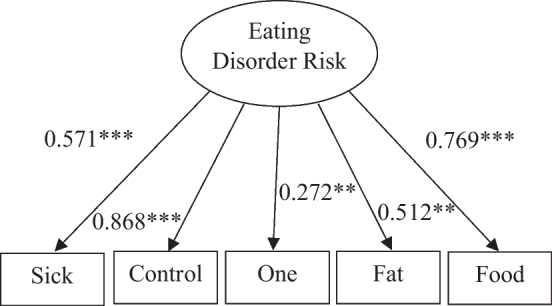
Path diagram for SCOFF. *p < 0.05 **p < 0.01 ***p < 0.001

In evaluating the unidimensionality of the construct, confirmatory factor analyses indicated acceptable model fit with a CFI value of 0.993, a TLI value of 0.985, and a RMSEA value of 0.029. The Chi-square (χ^2^) statistic was statistically significant, χ^2^(5) = 351.592, *p* < 0.001 indicating a lack of model fit but the Chi-square (χ^2^) statistic has been indicated as sensitive to sample size and model complexity. Overall, we consider model fit to be acceptable in reviewing all statistics. Figure 1 provides the path diagram with standardized path values for factor loadings, whereas values ranged from 0.272 to 0.868. All paths were statistically significant at the 0.05 level or less with level of statistical significance indicated on Fig. 1. The internal consistency of scores for the data obtained revealed a Cronbach’s alpha value of 0.556, which may be considered as low. After establishing unidimensionality, we next evaluated the local independence of items by comparing the model with and without residual terms being correlated after accounting for the shared variance of the latent construct. Table 2 also provides a summary of model comparison values including the AIC, BIC, and adjusted BIC values for local independence as well as comparing IRT models. For local independence, it appears that this assumption has been met as having lower AIC, BIC, and adjusted BIC values.

**Table 2:** Summary of model comparisons

	AIC	BIC	Adjusted BIC
Local independence			
Not Met	213,461.970	213,595.760	213,548.090
Met	213,181.560	213,324.270	213,273.420
Model			
2pL	350,610.870	350,703.990	350,672.210
3pL	350,620.890	350,760.560	350,712.890
4pL	350,462.890	350,649.140	350,585.580

**Fig. 2:**
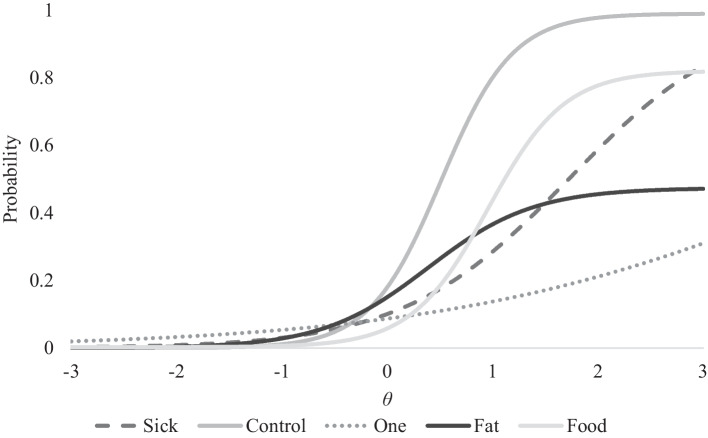
Item characteristic curves of the SCOFF

From examining these values on Table 2, it also appears that the 4pL model fit the data best as compared to the other models tested. From this 4pL IRT model, Table 3 provides a summary of item parameter values for each of the four parameters along with standard errors for each item. Item discrimination (*a*) values ranged from *a* = 0.517 (*SE* = 0.016) to *a* = 2.944 (*SE* = 0.129) indicating acceptable item discrimination. Item difficulty (*b*) values ranged from *b* = 0.383 (*SE* = 0.055) to *b* = 4.546 (*SE* = 0.133) indicating sufficient coverage of the construct. Item pseudo-guessing (*c*) parameter values ranged from *c* ≤ 0.001 (*SE* = 0.001) to *c* = 0.003 (*SE* = 0.001) indicating these values being consistently low and acceptable. Item pseudo-social desirability (*d*) parameter values ranged from *d* = 0.474 (*SE* = 0.024) to *d* = 1.00 (*SE* = 0.001) indicating that some items have not acceptable values. In particular, the Fat item (*d* = 0.474, *SE* = 0.024) and the Food item (*d* = 0.821, *SE* = 0.040) had lower than acceptable pseudo-social desirability values. Figure 2 provides the item characteristic curves for each item of the SCOFF. As for gender differences, Table 4 provides the item parameter estimates according to each group. There were an insufficient number of responses outside of the gender binary. There was no statistically significant (i.e., *p* < 0.050) differential item functioning across the parameters.

**Table 3:** Item parameter estimates for SCOFF items

	*a*	*b*	*c*	*d*
Item 1: Sick	1.270	1.722	0.000	1.000
*SE*	(0.019)	(0.019)	0.001	0.001
Item 2: Control	2.944	0.510	0.000	0.990
*SE*	(0.129)	(0.019)	0.001	0.130
Item 3: One	0.517	4.546	0.000	1.000
*SE*	(0.016)	(0.133)	0.001	0.001
Item 4: Fat	1.986	0.383	0.000	0.474
*SE*	(0.183)	(0.082)	0.001	0.024
Item 5: Food	2.749	0.383	0.003	0.821
*SE*	(0.231)	(0.055)	0.001	0.040

**Table 4:** Item parameter estimates for SCOFF items

	*a*		*b*		*c*		*d*	
	Women	Men	Women	Men	Women	Men	Women	Men
Item 1: Sick	1.304	1.167	1.605	2.079	< 0.001	< 0.001	1.000	1.000
*SE*	0.023	0.036	0.021	0.048	< 0.001	< 0.001	< 0.001	< 0.001
Item 2: Control	2.850	2.92	0.341	0.975	< 0.001	< 0.001	0.987	0.997
*SE*	0.193	0.173	0.029	0.028	< 0.001	< 0.001	0.019	0.017
Item 3: One	0.550	0.60	4.441	3.545	< 0.001	< 0.001	1.000	0.897
*SE*	0.021	0.031	0.153	0.172	< 0.001	< 0.001	< 0.001	0.014
Item 4: Fat	1.810	2.585	0.234	0.613	< 0.001	< 0.001	0.490	0.391
*SE*	0.214	0.557	0.109	0.147	< 0.001	< 0.001	0.032	0.040
Item 5: Food	2.924	2.903	0.769	1.341	< 0.001	0.017	0.793	0.765
*SE*	0.176	0.749	0.048	0.176	< 0.001	0.007	0.031	0.128

ROC curve results indicated that SCOFF scores sufficiently screened for eating disorders based upon self-reported diagnoses, AUC = 0.779 (*SE* = 0.004), *z* = 61.078, *p* < 0.001. A sensitivity value of 70.66 was achieved along with a corresponding specificity value of 74.67. A weighted SCOFF scoring procedure revealed slightly better model fit, AUC = 0.783 (*SE* = 0.004), *z* = 61.443, *p* < 0.001. This weighted procedure weighted the two items that had lower than acceptable fourth parameter values (i.e., the Fat and Food items). With this new scoring of the SCOFF, a sensitivity value of 65.941 was achieved along with a corresponding specificity value of 78.413. This AUC value for the weighted score was significantly better, ΔAUC = 0.004, *SE* = 0.0008, *z* = 4.813, *p* < 0.001 but practically it was only slightly better.

## Discussion

Results of the current study suggest evidence that the SCOFF is psychometrically sufficient overall in terms of construct and criterion validity in view of confirmatory factor analyses, item response theory analyses, and ROC curve analyses. Values for the first three parameters of item discrimination, item difficulty, and item pseudo-guessing were all acceptable across items of the SCOFF. However, the IRT analyses suggest that the fourth parameter of the upper asymptote indicating some degree of pseudo-social desirability identified two items of the SCOFF (i.e., fat and food items) that could have performed better. To reiterate, the fourth item of fat stated, “Do you believe yourself to be **F**at when others say you are too thin?” [38], p. 1467). We should note that the word, fat can be an emotionally reactive term, thus it is not surprising that this item would prompt a sense of perceived stigma and that respondent would respond in a socially desirable way. Alternatively, individuals may simply not consider fat as the appropriate word but rather ‘not thin enough.’ The other item was the fifth item of food, which stated, “Would you say **F**ood dominates your life?” [38], p. 1467). While the word food may be a neutral term, the use of the verb, ‘dominates’ can elicit a defensive responsive to the claim that one’s eating disorder would dominate one’s life. Again, individuals perceiving the stigma of this domination may respond in a socially desirable way denying it.

Alternative explanations may exist for these patterns of responding that may be as simple as confusion over the word(s) or a different understanding of the meaning of an item as can be the case with academic achievement items [6]. For instance, individuals who may desire control over their lives hence the association between obsessive compulsive tendencies and eating disorders [28, 35]. These individuals may object or not understand the idea of food dominating their life (i.e., ‘Would you say Food dominates your life?’). In fact, these individuals may view it as they are dominating food. As for the item of “Do you believe yourself to be **F**at when others say you are too thin?,” there are individuals who may find this item confusing as there are no persons in their life that say they are too thin, either due to social isolation or enabling behaviors of family and friends [2, 36].

As for the ROC curve analyses, these results indicate the SCOFF as an acceptable screening instrument for eating disorders as self-reported by individuals. This self-report by individuals presents a degree of subjectivity in screening and diagnosis. The SCOFF may be considered promising in its ability to screen for eating disorders with only five, dichotomous response format items. The weighted SCOFF performed slightly better but there appears to have been a trade-off between sensitivity versus specificity. The original SCOFF scores revealed higher sensitivity and lower specificity values while the weighted SCOFF scores revealed lower sensitivity but higher specificity values. As screening instruments typically privilege sensitivity over specificity [31], it is understandable that any increased overall performance from weighted SCOFF scores may not be worth implementing.

### Limitations and considerations

We should note several limitations that should be considered when evaluating the results of the current study. First, self-reported diagnoses of eating disorders, which may include both lifetime and current statuses, were utilized for the ROC curve analyses may be limited in their generalizability to and across clinical settings. This utilization of self-reported diagnoses does lend itself to being patient-centered despite having an unclear timeframe. However, self-reported diagnoses can be conflated with stigma and social desirability such that individuals may not want to disclose this information but the survey was anonymous and involved no face-to-face contact. Furthermore, individuals may under-recognize their eating disorder symptoms and may not be as likely to seek diagnosis or services from health care professionals. Additionally, the sample utilized was exclusively of young adults who were in college settings as students. The mean age of the sample was approximately 23 years old. Results consequently may only be generalizable to other similar young adult populations rather than the general population [30, 44, 45] or clinical populations [13, 33, 39].

Second, the internal consistency of scores for the data obtained was low despite evidence for construct validation being quite high in examining for unidimensionality via confirmatory factor analyses. Third, Jin [27] found limited evidence as to the efficacy of screening for eating disorders in general due to a limited number of research studies in this area. This finding from Jin [27] should not be confused to mean that screening instruments do not psychometrically work but rather that they have not been used enough in the process leading to treatment or intervention to indicate efficacy. This criticism is not exclusive to the SCOFF by any means. Fourth, Pica may be considered a distinct form of eating disorder, which may present as a limitation to the current study. Additionally, results may not necessarily generalize to this population of the individuals given the number of participants with Pica who were included in the current study was quite small at less than one percent. Fifth, there is a degree of subjectivity in screening for any disorder that should be noted, which then in turns introduces a degree of subjectivity as to diagnosis that may be heightened when considering self-reported diagnoses. Finally, the current study was not a comprehensive examination of all forms of reliability and validity as the scope of the study was limited to item response theory and ROC curve analyses given the presence of extant psychometric research [3, 8, 32, 42, 48].

### Strengths

The community-based and non-clinical nature of the sample may be considered a strength of the study in terms of being generalizable to the population. The Healthy Minds Study included participants across seventy-five institutions of higher education [25] in the effort to produce a nationally representative sample. Beyond the sample characteristics, the study represents the first four parameter logistic (4pL) item response theory examination of the SCOFF. All other item response theory examinations of the SCOFF were limited to two parameter logistic models [9] for a high school sample [10], for a seventh grade sample). In both of these studies, the item discrimination (*a*) values were lower across all the items (*Ma* = 1.474 in [9] and *Ma* = 1.591 in [10] while the average item discrimination values for the current study were better at *Ma* = 1.893. As for item difficulties, the ranges of item difficulty values were similar for Bean [10] with a range of 4.211 and the current study having a range of 4.163. However, the range of item difficulties values was much smaller in Bean [9] with a range of 1.141. Limiting to a two-parameter logistic (2pL) model is not unreasonable given the very low, non-existent third parameter logistic (3pL) estimates, so it is logical that researchers would not then proceed to explore a 4pL model. A 4pL model, however allows us to examine the non-endorsement of behavioral items of individuals who are otherwise endorsing similar items at a high frequency. In this way, we can detect patterns of item response that may be counter to their other responses to other items.

### Future research

Future research should consider how the presence of comorbid or co-ocurring conditions may influence SCOFF scores as well as the self-report of eating disorders. It would be interesting to examine how SCOFF scores differ according to the comorbid profile among individuals with self-reported eating disorders. Additionally, future research should consider changing diagnostic criteria in eating disorders, which can also contribute to issues with detection and diagnosis [30]. Finally, future research should further delve into the meaning of individuals with otherwise high scores on the SCOFF not endorsing an item related that is clearly related to a symptom of an eating disorder. In the current study, we termed this behavior as the result of the perceived stigma experienced by individuals who then in turn respond in what they considered a socially desirable way.

In conclusion, the results of the current study suggest evidence of the sufficient psychometric properties of the SCOFF in terms of confirmatory factor and item response theory analyses. However, two items of the SCOFF revealed that individuals who otherwise endorsed other items of the SCOFF were less likely to endorse the items of Fat and Food. It is hypothesized that this is the result of perceived stigma regarding those two items that prompts individuals to respond in a socially desirable way. A weighted scoring procedure was developed to counteract the performance of these two items, but the psychometric performance was only slightly better and there would be a clear tradeoff of specificity over sensitivity if utilized. Future research should consider other ways to counteract such perceived stigma.

### What is already known on this subject?

As young adults, college students represent a population at-risk for developing eating disorders. The SCOFF is a screening instrument that has been used to detect eating disorders early on. Detecting eating disorders is important for treatment.

### What does this study add?

We examined the SCOFF with a sample of college students. The results indicate people were less likely to respond as expected on two items of the SCOFF on Fat and Food. Stigma and social desirability were suggested as reasons for this.
